# Perceptions of the Diabetes Online Community’s Credibility, Social Capital, and Help and Harm: Cross-Sectional Comparison Between Baby Boomers and Younger Adults

**DOI:** 10.2196/10857

**Published:** 2019-09-26

**Authors:** Michelle L Litchman, Linda S Edelman

**Affiliations:** 1 University of Utah College of Nursing Salt Lake City, UT United States

**Keywords:** diabetes mellitus, online social networking, social capital, trust, social media, older adult

## Abstract

**Background:**

The use of online health communities such as the diabetes online community (DOC) is growing. Individuals who engage in the DOC are able to interact with peers who have the same medical condition. It is not known if older adults are perceiving the DOC differently compared with younger adults.

**Objective:**

The purpose of this study was to explore and understand how the DOC is perceived in terms of social capital, source credibility, and help and harm. The findings from this study will shed light on how users of different age groups (baby boomers and younger adult counterparts) perceive DOC use.

**Methods:**

This study represents a subset of participants from a larger study of DOC users. Baby boomers and younger adults with diabetes were recruited from the DOC to participate in a cross-sectional survey. Demographics, electronic health use (reasons to join the DOC, DOC intensity, DOC engagement, internet social capital, and help or harm from the DOC), source credibility, health-related quality of life, and diabetes self-care data were collected. We examined the differences between baby boomer and younger adult responses.

**Results:**

The participants included baby boomers (N=76) and younger adult counterparts (N=102). Participants scored their diabetes health care team (mean 33.5 [SD 8]) significantly higher than the DOC (mean 32 [SD 6.4]) with regard to competence (*P*<.05) and trustworthiness (diabetes health care team mean 36.3 [SD 7.1]; DOC mean 33.6 [SD 6.2]; *P*<.001). High bonding and bridging social capital correlated with high DOC intensity (*r*=.629; *P*<.001 and *r*=.676; *P*<.001, respectively) and high DOC engagement (*r*=.474; *P*<.01 and *r*=.507; *P≤*.01, respectively). The greater majority (69.8%) reported the DOC as being helpful, and 1.8% reported that the DOC had caused minor harm. Baby boomers perceived DOC credibility, social capital, help, and harm similarly to their younger adult counterparts.

**Conclusions:**

Baby boomers are using and perceiving the DOC similarly to younger adults. DOC users find the DOC to be credible; however, they scored their health care team higher with regard to competence and trustworthiness. The DOC is beneficial with low risk and may augment current diabetes care.

## Introduction

### Background

Diabetes is a complex chronic condition that requires ongoing attention to day-to-day activities to achieve adequate glucose management. Individuals with diabetes are expected to spend more than 2 hours per day carrying out recommended self-care [1]. The time and intensity required for self-care, the complexities of diabetes, and the often unavoidable health fluctuations associated with their condition can be physically and emotionally taxing. Therefore, adequate informational and emotional support is imperative for patients to effectively manage their diabetes [2-4].

There has been a paradigm shift in which the patient’s role has elevated from a passive recipient to an active consumer of health care [5]. As active consumers, patients are seeking more information to assist them in making decisions about their health. This is particularly true for individuals with chronic conditions who need information that will allow them to be successful in long-term disease management. Access to the internet has provided consumers with a myriad of easily accessible health information resources to support health care decisions, including social networks such as diabetes online communities (DOCs).

DOCs are grassroots digital locations in which individuals affected by diabetes interact, educate, and offer support to peers [6,7]. DOC use is associated with self-care, quality of life, and better glycosylated hemoglobin (HbA_1c_) [7]. Older adults, specifically, have found DOCs to be useful in improving knowledge about self-care and receiving emotional support [6,8].

The prevalence of, and proportion of, older adults with diabetes is simultaneously increasing. Baby boomers (born from 1946 to 1964), responsible for the vast growth of older adults, have a higher incidence of chronic conditions such as diabetes [9,10]. With the oldest baby boomer turning 65 years of age in 2011, the number of older adults with diabetes is rapidly increasing. Online health information seeking, such as DOC use, has been viewed as helpful in providing secondary prevention [11]. Baby boomers are the first generation to age using the internet and social media. Importantly, the adoption of online health communities is on the rise for older adults [12,13]. Therefore, it is important to determine if baby boomers perceive the credibility of online communities differently than their younger counterparts as this may inform online community use as they age.

One way that individuals establish credibility of online health information is through the guidance of peers as suggested in the Apomediation theory. The Apomediation theory proposes that individuals can bypass the traditional hierarchical medical system and, through a filtering process, collaborate with experienced peers who guide them toward credible and relevant information [14]. As it relates to diabetes, individuals may be more reliant on their health care providers upon initial diagnosis. However, over time, autonomy, knowledge, and self-efficacy are gained, allowing for interaction with experienced peers. Source credibility is one of the domains identified in the Apomediation theory [14,15] and will be measured in this study.

### Source Credibility

Health information is not credible without trust in the message and source. Source credibility, defined as a characteristic that helps readers determine if information is believable, is associated with perceptions of competence, trustworthiness, and goodwill/caring [16]. Research suggests that online health community users are more likely to perceive community information as credible if it is based on firsthand knowledge of living with a health issue [17]. Furthermore, source credibility has been associated with emotional support in online health communities [18]. Although source credibility in peers may develop through the exchange of personal information and shared experiences, source credibility may be more difficult to ascertain in online environments because of reliance on text without the support of nonverbal cues and facial expressions [18,19].

### Social Capital

Participating in online health communities improves social capital [20]. Social capital, a term coined by Putnam [21], comprises the social connections, networks, and trust that allow individuals to work together as a community. There are 2 types of social capital. Bonding social capital includes close family and friends and is exclusive. Bonding social capital promotes group cohesion and social support. Bridging social capital is inclusive and is made up of heterogeneous networks of connections with weak ties. Bridging social capital allows for diffusion of information and diverse perspectives [22]. A number of studies have found an association between social capital, health and mortality [23-27]. Among those with chronic conditions, including diabetes, having a large network of social connections is associated with better self-management, physical and mental well-being, and coping [28]. Lack of social capital has been identified as a barrier in diabetes self-management [3]. Little is known about source credibility or social capital as it relates to peer health in the online context.

### The Diabetes Online Community Providing Help or Harm

It is unclear if individuals perceive DOC use as being helpful or harmful. The Pew Internet and Life Project found that 30% of US adults report that they or someone they know had been helped by following the advice or health information found online and only 3% reported being harmed [29]. In contrast, there is a potential for harm, such as inaccurate health information being reported and followed, public displays of unhealthy behaviors, and psychological impact from accessing offensive or biased content [30]. Therefore, it is important to evaluate how DOC users perceive information shared on the DOC as it relates to being helpful or harmful.

Despite the growing reach of the DOC, there is limited research focused on social capital, source credibility, and whether or not the DOC is helpful or harmful. The overarching objective of this study was to explore how the DOC is perceived as it relates to social capital, source credibility, and harm by way of age groups. Specifically, we aimed to understand the differences between younger adults (born between 1965 and 1996) and baby boomers (born between 1946 and 1964). The findings from this study will illuminate the possible benefits and disadvantages to DOC use.

## Methods

### Sample and Setting

The study sample was from a larger study of DOC users (N=183). Participants were eligible for the parent study if they were aged 18 years or older, had a diagnosis of diabetes (type 1, type 2, or latent autoimmune diabetes of adulthood), and could read English. Anyone identifying as a minor, caregiver for someone with diabetes, or having gestational diabetes was not included. Participants were recruited by posting information about the study with a link to the survey. Key opinion leaders shared the survey to support snowball sampling. The full sample included 183 adult DOC users. The participants completed a 129-item online survey using Research Electronic Data Capture (REDCap) software (Nashville, TN). A subsection of baby boomers completed an interview. The study procedures were approved by the University of Utah Institutional Review Board and the respective DOC administrators (TuDiabetes and Diabetic Connect). Previous research from the parent study has been published elsewhere [6,7].

This study focused on 176 DOC users categorized as baby boomers or younger adult counterparts. Participants born in 1945 or earlier were not included in this study (N=5).

### Measures

This paper will examine online survey results from the parent study survey including demographics specific to baby boomers compared with younger adult counterparts, electronic health use (reasons to join the DOC, DOC intensity, DOC engagement, internet social capital, and perceived help or harm from the DOC), source credibility, HRQOL (health related quality of life), and diabetes self-care. Details for each measure are noted below.

#### Demographics

Social and demographic data included 11 items focused on gender, marital status, education level, employment, annual household income, age, ethnicity, race, country and state, living setting, and insurance status.

#### Health History

Self-reported health history data included 8 items focused on diabetes type, length of diabetes duration in years, current diabetes treatments, most recent HbA_1c_ level, type of medical practice and provider used for diabetes care, frequency of diabetes provider visits, and diabetes-related complications.

#### Electronic Health Use

Twenty-two items were collected on how participants navigate the DOC and if the participants’ health care provider supported their DOC use.

#### Reasons to Join the Diabetes Online Community

Participants were asked to identify reasons why someone with diabetes should join the DOC. Thirteen items were developed based on an anecdotal dLife (Diabetes Life) article [31].

#### Diabetes Online Community Intensity

The DOC Intensity Scale is an 8-item tool adapted from the Facebook Intensity Scale [32] to measure how often and for how long individuals engaged in the DOC and to determine emotional connectedness and integration into daily activities. Scores range from 0 to 5 with higher scores indicating more DOC intensity. The Cronbach coefficient for DOC intensity was .85.

#### Diabetes Online Community Engagement

The DOC Engagement Scale is a 5-item tool developed by the authors and informed by qualitative analysis [33] to measure engagement or interaction with other DOC users. Specifically, this tool was used to measure whether or not participants shared clinical information, requested or provided clinical guidance or feedback, or received or provided emotional support. Scores range from 0 to 5 with higher scores indicating more DOC engagement. The Cronbach coefficient for DOC engagement was .73.

#### Internet Social Capital Scale

The Internet Social Capital Scale is designed to measure bonding (10 items) and bridging (10 items) social capital in both online and offline populations using a 5-point Likert scale [34] such as DOC use. The Likert response scale ranges from strongly agree to strongly disagree. The terms *offline* and *online*, which can be used interchangeably based on the study population, were replaced with *DOC* in this study. There were 3 questions from the Internet Social Capital Scale bonding subscale that did not pertain to the study population. The question “If I needed an emergency loan of $500, I know someone online that I can turn to” was changed to “If I needed an emergency loan of diabetes supplies, I know someone on the DOC I can turn to.” The questions “The people I interact with on the DOC would put their reputation on the line for me” and “The people I interact with on the DOC would be good job references for me” were omitted from the survey. Permission was obtained from Williams [34] to use and adapt the Internet Social Capital Scale for this study. The adapted 7-item bonding social capital scale and 10-item bridging social capital scale each have possible scores of 0 to 5; higher scores indicate higher levels of social capital. In this study, the Internet Social Capital Scale will measure DOC bonding and bridging social capital.

#### Help and Harm

Overall, 2 questions were asked related to perceived help and harm, asking participants if they, or anyone they knew, had been helped or harmed by following advice or health information found on the DOC. Responses included major help, moderate help, minor help, no help, or do not know. Responses were then dichotomized into yes and no responses with regard to any help or harm, or no help or harm.

#### Health-Related Quality of Life

The SF12-v2 (short form 12-item version 2) is a 12-item tool used to measure physical and mental health status. A 4-week recall was used in this study. Norm-based scoring (mean 50 [SD 10]) was used for this analysis [35]. The Cronbach coefficient for the SF-12v2 was .88 (physical=.77 and mental=.86).

#### Diabetes Self-Care

The Self-Care Inventory Revised (SCI-R) is a 15-item tool to measure diabetes self-care behaviors and can accommodate natural variation in treatment plans for patients with type 1 and type 2 diabetes. The scores range from 0 to 100 [36]. The Cronbach coefficient for the SCI-R was .68.

#### Source Credibility

The revised source credibility scale [16] was used to measure how participants viewed the credibility of the diabetes health care team and the DOC. The scale includes 18 items measuring 3 factors—competence, trustworthiness, and goodwill/caring—using a 7-point semantic differential scale. The Cronbach alpha scores range from .85 to .92 when looking at the dimensions separately and .94 when scored as a single measure. This scale was used twice in this study; first, to measure how participants rated the source credibility of their diabetes health care team. The diabetes health care team included anyone who cared for the patient’s diabetes. Second, it was also used to measure how participants rated the source credibility of the DOC. Possible scores ranged from 0 to 42.

### Analysis

A participant code was assigned to all survey responses, and all data were maintained in REDCap [37]. Data were screened, and multiple entries were cleaned accordingly. Missing data were imputed with the appropriately scaled item means in the calculation of total scores for the validated scales in accordance with standard scoring methods. All other missing data were excluded pairwise. There were less than 10% of missing data for each analysis.

Statistical analysis was performed using SPSS 21 (New York City, New York) [38]. Analyses were conducted to determine the relationships between and interactions among demographic variables, source credibility, social capital, help, and harm. These analyses included correlations, independent and 1-sample *t* tests, and analyses of variance (followed by LSD (least significant difference)-adjusted post hoc tests, where appropriate). For inference, the alpha was set at .05.

## Results

### Participant Characteristics

A total of 178 participants met the criteria for this study: 43% were baby boomers and 57% were younger adults. Overall, the participants were more likely to be female, white, living in the United States, educated with a college degree, and insured and have type 1 diabetes. Baby boomers were more likely to be living in the United States and more likely to have type 2 diabetes or latent autoimmune diabetes of adulthood (LADA) compared with younger adult counterparts. There were no significant differences between baby boomers and younger adult counterparts regarding gender, education level, income, or presence of insurance (see Table 1).

### Diabetes Online Community Source Credibility

The mean DOC competence score was 31.9 (SD 6.5), the mean DOC caring/goodwill score was 31.9 (SD 7.2), and the mean DOC trustworthiness score was 33.6 (SD 6.3). Each factor score had a possible range of 0 to 42. The Cronbach coefficients for the DOC source credibility scale were DOC competency, alpha=.89; DOC caring/goodwill, alpha=.89; and DOC trustworthiness, alpha=.91.

DOC source credibility (competency, caring/goodwill, and trustworthiness) positively correlated with diabetes self-care, DOC intensity, DOC engagement, and bonding and bridging social capital as detailed in Table 2. DOC competence scores were higher (*P*<.05) for individuals who had told their health care providers about their DOC use and felt supported to continue doing so (mean 34.3 [SD 6.1]) than those who were not sure if their health care providers supported their DOC use because they had not told their health care providers about it (mean 31 [SD 6.6]). Similarly, all participants reported higher DOC caring/goodwill scores if they had told their health care providers about their DOC use and their health care providers supported it (mean 34.7 [SD 5.4]; *P*<.01) or were not sure if their providers supported their DOC use even after they had reported it (mean 34.2 [SD 7.4]; *P*<.05) when compared with those who had not told their health care providers about their DOC use at all (mean 30.8 [SD 7.4]). DOC source credibility factor scores were not related to age, gender, diabetes type, diabetes duration, diabetes treatment, diabetes-related complications, HbA_1c_, or health-related quality of life. There were no significant differences for DOC source credibility factors (competence, caring/goodwill, or trustworthiness) between baby boomers and younger adult counterparts.

**Table 1:** Demographics of study participants, N=178.

Variable		Baby boomers^a^	Younger adult counterparts^b^	Total	*P* value
Age (years), mean (SD); range		—^c^	—	43.8 (13.2); 18-67	—
**Gender, n (%)**					**.18^d^**
	Female	51 (67.1)	77 (75.4)	128 (71.9)	—
	Male	24 (31.6)	23 (22.5)	47 (26.4)	—
**Race, n (%)**					**.65^d^**
^ ^	American Indian or Alaskan Native	0 (0)	2 (2.0)	2 (1.1)	—
	Asian	1 (1.3)	2 (2.0)	3 (1.7)	—
	African American	1 (1.3)	1 (1.0)	2 (1.1)	—
	White	73 (97.3)	96 (93.1)	169 (94.9)	—
**Country, n (%)**					**.025^d^**
	United States	69 (90.7)	78 (76.5)	147 (82.6)	—
	Not United States	7 (9.2)	23 (22.5)	30 (16.9)	—
**Education, n (%)**					**.69^d^**
	Some high school	0 (0)	2 (2.0)	2 (1.1)	—
	High school graduate	5 (6.6)	5 (4.9)	10 (5.6)	—
	Some college	14 (18.4)	13 (12.7)	27 (15.2)	—
	Associate’s degree	9 (11.8)	11 (10.8)	20 (11.2)	—
	Bachelor’s degree	25 (32.9)	39 (38.2)	64 (36)	—
	Gradate or professional degree	23 (30.3)	31 (30.4)	54 (30.3)	—
**Insurance, n (%)**					**.062^e^**
	Insured	73 (96.1)	84 (82.4)	157 (82.6)	—
	Uninsured	2 (2.6)	9 (8.8)	11 (16.9)	—
**Diabetes type, n (%)**					**.007^d^**
	Type 1	45 (59.2)	82 (80.4)	127 (71.3)	—
	Type 2	18 (23.7)	13 (12.7)	31 (17.4)	—
	Latent autoimmune diabetes of adulthood	13 (17.1)	7 (6.9)	20 (11.2)	—

^a^n=76 (42.7%).

^b^n=102 (57.3%).

^c^Not applicable.

^d^Chi-square test.

^e^Fisher exact test.

**Table 2:** Correlations for diabetes online community source credibility, N=178.

Variable	Diabetes online community (DOC)		
	Competence	Caring/goodwill	Trustworthiness
Diabetes self-care	.144	.158^a^	.169^a^
DOC intensity	.364^c^	.465^c^	.322^c^
DOC engagement	.196^b^	.285^c^	.215^b^
Bonding social capital	.368^c^	.504^c^	.412^c^
Bridging social capital	.369^c^	.484^c^	.380^c^
Physical HRQOL^d^	−.002	.040	.060
Mental HRQOL	−.021	.014	.034

^a^*P*<.05.

^b^*P*<.01.

^c^*P*<.001.

^d^HRQOL=health related quality of life.

### Diabetes Health Care Team Source Credibility

The 3 factors of source credibility were also measured to determine the credibility of information coming from the participants’ diabetes health care provider team. The mean score for diabetes health care provider team competence was 29.8 (SD 5.5), caring/goodwill was 32.8 (SD 9.1), and trustworthiness was 36.1 (SD 7.4). The Cronbach alpha values for the diabetes health care team were as follows: competence, alpha=.90; caring/goodwill, alpha=.95; and trustworthiness, alpha=.93.

The relationships were identified between DOC and diabetes health care team source credibility scores (see Table 3). DOC competence and trustworthiness positively correlated with diabetes health care team trustworthiness. There were no relationships between DOC caring/goodwill and diabetes health care team competence or caring/goodwill.

Baby boomers (mean 34.61 [SD 9.0]; *P*<.05) found their diabetes health care provider team as having more caring/goodwill than younger adult counterparts (mean 31.46 [SD 9.0]). There were otherwise no significant differences between groups with regard to diabetes health care provider competence or trustworthiness factors.

There were differences in how all participants scored source credibility when comparing the DOC and their health care provider team. Participants scored their diabetes health care team (mean 33.5 [SD 8]) significantly higher than the DOC (mean 32 [SD 6.4]) with regard to competence (*P*<.05) and trustworthiness (diabetes health care team mean 36.3 [SD 7.1]; DOC mean 33.6 [SD 6.2]; *P*<.001). There was no statistically significant difference in how participants scored DOC and diabetes health care team caring/goodwill.

There were similarities and differences in how DOC and diabetes health care team source credibility were associated with diabetes self-care, DOC intensity, DOC engagement, bonding and bridging social capital, and health-related quality of life (see Table 4). DOC and diabetes health care team source credibility were similar in that all source credibility factors correlated with diabetes self-care. Conversely, although DOC source credibility was associated with DOC intensity, DOC engagement, and bonding and bridging social capital, diabetes health care team source credibility correlated with health-related quality of life.

**Table 3:** Pearson product correlations between diabetes online community and diabetes health care team source credibility, N=178.

Variable	Diabetes online community		
	Competence	Caring/goodwill	Trustworthiness
Diabetes health care team competence	.098	.115	.148
Diabetes health care team caring/goodwill	.152^a^	.119	.140
Diabetes health care team trustworthiness	.257^b^	.137	.270^c^

^a^*P*<.05.

^b^*P*<.01.

^c^*P*<.001.

**Table 4:** Pearson product correlations for diabetes health care team source credibility, N=178.

Variable	Diabetes health care team		
	Competence	Caring/goodwill	Trustworthiness
Diabetes self-care	.188^a^	.176^a^	.195^a^
Diabetes online community (DOC) intensity	.038	.008	.054
DOC engagement	.117	.128	.148
Bonding social capital	.000	−.012	.002
Bridging social capital	.124	.114	.129
Physical HRQOL^b^	.214^c^	.234^c^	.195^a^
Mental HRQOL	.268^d^	.340^d^	.247^d^

^a^*P*<.05.

^b^HRQOL=health related quality of life.

^c^*P*<.01.

^d^*P*<.001.

### Social Capital

The Internet Social Capital Scale bonding mean score was 3.08 (SD 0.64) and bridging mean score was 3.68 (SD 0.68). The Cronbach coefficient for the Internet Social Capital Scale was .89 (bonding=.69 and bridging=.92). High bonding and bridging social capital correlated with high DOC intensity (*r*=.629; *P*<.001 and *r*=.676; *P*<.001, respectively) and high DOC engagement (*r*=.474; *P*<.01 and *r*=.507; *P*<.01, respectively; see Table 5). Furthermore, high bonding (*P*<.001) and bridging (*P*<.001) social capital was identified in those who reported *yes* to all 13 reasons to join a DOC (see Table 6). Bonding (*P*<.001) and bridging (*P*<.001) social capital scores were higher in those who had told their health care provider about their DOC use and felt supported (bonding mean 3.5 [SD 0.63]; bridging mean 4.2 [SD 0.51]) or were not sure (bonding mean 3.26 [SD 0.57]; bridging mean 3.93 [SD 0.48]) than those who had never told their health care providers about their DOC use at all (bonding mean 2.94 [SD 0.59]; bridging mean 3.48 [SD 0.68]). There was a negative correlation between bonding social capital and age (*r*=−.200; *P*<.01).

**Table 5:** Correlation matrix for health indicators, N=178.

Indicator	1	2	3	4	5	6	7
Diabetes online community (DOC) intensity	1.00	—^a^	—	—	—	—	—
DOC engagement	.572^b^	1.00	—	—	—	—	—
Physical HRQOL^c^	−.043	.102	1.00	—	—	—	—
Mental HRQOL	−.076	.074	.651^b^	1.00	—	—	—
Bonding social capital	.629^b^	.474^b^	.022	.028	1.00	—	—
Bridging social capital	.676^b^	.507^b^	−.010	−.014	.679^b^	1.00	—
Diabetes self-care	.236^d^	.170^e^	.097	.301^d^	.127	.234^d^	1.00

^a^Not applicable.

^b^Significance at the <.001 level.

^c^HRQOL=health related quality of life.

^d^Significance at the <.01 level.

^e^Significance at the <.05 level.

**Table 6:** Diabetes online community users who reported diabetes online community benefits and its relationship to bonding and bridging social capital, N=169–176.

Diabetes online community (DOC) benefit		Bonding social capital		Bridging social capital	
		Mean (SD)	*P* value	Mean (SD)	*P* value
**Feel understood**					
	Yes	3.2 (0.61)	<.001	3.9 (0.53)	<.001
	No	2.6 (0.53)	<.001	2.9 (0.64)	<.001
**Feel less alone**					
	Yes	3.2 (0.62)	<.001	3.9 (0.55)	<.001
	No	2.7 (0.48)	<.001	3.1 (0.71)	<.001
**Feel more empowered**					
	Yes	3.3 (0.62)	<.001	3.9 (0.53)	<.001
	No	2.6 (0.50)	<.001	3.0 (0.62)	<.001
**Feel support through rough times**					
	Yes	3.3 (0.58)	<.001	4.0 (0.45)	<.001
	No	2.7 (0.58)	<.001	3.3 (0.71)	<.001
**Learn new diabetes management strategies**					
	Yes	3.2 (0.63)	.001	3.8 (0.58)	<.001
	No	2.7 (0.63)	.001	3.1 (0.77)	<.001
**Learn research and treatment alternatives**					
	Yes	3.2 (0.63)	<.001	3.8 (0.60)	<.001
	No	2.7 (0.56)	<.001	3.0 (0.76)	<.001
**Get answers to diabetes questions**					
	Yes	3.2 (0.63)	<.001	3.8 (0.56)	<.001
	No	2.7 (0.53)	<.001	3.1 (0.73)	<.001
**Learn about potential side effects of drugs or devices**					
	Yes	3.2 (0.62)	<.001	3.9 (0.59)	<.001
	No	2.8 (0.63)	<.001	3.4 (0.75)	<.001
**Learn things that my health care provider did not know**					
	Yes	3.2 (0.63)	<.001	3.9 (0.60)	<.001
	No	2.9 (0.61)	<.001	3.4 (0.72)	<.001
**Learn strategies to improve insurance coverage for diabetes related medications, supplies, or tools**					
	Yes	3.8 (0.63)	<.001	3.9 (0.62)	<.001
	No	2.8 (0.55)	<.001	3.5 (0.67)	<.001
**Discussed a topic learned from DOC with my health care provider**					
	Yes	3.3 (0.63)	<.001	3.9 (0.57)	<.001
	No	2.9 (0.58)	<.001	3.5 (0.68)	<.001
**Help others**					
	Yes	3.2 (0.61)	<.001	3.8 (0.54)	<.001
	No	2.6 (0.57)	<.001	3.0 (0.87)	<.001

### Help

The greater majority of DOC participants (69.8%) reported that they or someone they knew were helped by following advice or health information on the DOC; although, 27.3% were not sure. Those with type 1 diabetes (count 88, expected count 84.4) or LADA (count 16, expected count 14) were more likely to report help from the DOC when compared with those with type 2 diabetes (count 16, expected count 21.6; *P*<.05). There were differences in DOC source credibility scores for caring/goodwill and levels of help from the DOC: *F*_2,166_=5.29; *P*<.01. Those who reported that the DOC provided any level of help (mean 33.1 [SD 6.2]) had higher DOC caring/goodwill scores than those who reported “don’t know” (mean 29.3 [SD 8.8]). There were no significant differences in the perception of the DOC being helpful among baby boomers or younger adult counterparts.

### Harm

There was a very small percentage (1.8%) of participants who reported that they or someone they knew had been harmed, with the degree being minor, by following the advice or health information found on the DOC. Nearly half (45%) of the DOC participants reported that they did not know if harm had taken place. Participants had higher DOC competence scores if they reported no harm (mean 33.1 [SD 6.2]) than those who reported “don’t know” (mean 30.7 [SD 6.7]; *F*_2,166_=3.53; *P*<.05) and had higher DOC caring/goodwill scores if they reported no harm (mean 33.3 [SD 6]) than those who reported “don’t know” (mean 30.7 [SD 8.2]; *F*_2,165_=3.67; *P*<.05). Furthermore, participants had higher DOC trustworthiness scores if they reported no harm (mean 35 [SD 5.8]) than those who reported “don’t know” (mean 32.4 [SD 6.6]; *F*_2,161_=4.3; *P*<.05). There were no significant differences in the report of being harmed by gender or diabetes type. In addition, there were no significant differences in perception of the DOC being harmful among baby boomers or younger adult counterparts.

## Discussion

### Principal Findings

This is the first study known by the authors to examine differences in how baby boomer and younger adult counterparts perceive the DOC in terms of social capital, source credibility, and help and harm. Below, we discuss the significant findings and the implications for clinical practice.

Despite considerable differences generationally, in this study, baby boomers and younger adult DOC users perceived the credibility of DOC information similarly. Baby boomers are the first generation to transition into older adulthood with internet skills. Positioned as digital natives, baby boomers had to learn computer, internet, and social media skills later in life, opposed to digital natives or younger adults, who grew up with these technologies. Studies have shown that baby boomers are more trusting of online health information compared with older adults [39], which may explain why there was no difference between age groups. Baby boomers will seek out the internet first with health-related questions [40], which may be one way for baby boomers to be able to find DOCs.

Although there have been documented benefits to DOC use [7,41,42], baby boomers, in particular, may experience other benefits not yet explored. Research indicates isolation and loneliness is an increasing concern in older adult populations [43,44]. One solution to mitigate feelings of seclusion in older adults with diabetes is DOC use. In a semantic analysis of 1 DOC, TuDiabetes, Lewis et al found that older adults with type 2 diabetes used DOCs for companionship and support [45]. A content analysis of DOC users on Twitter found that participants anticipated that they would continue using the DOC into old age, maintaining lifelong connections with peers they interact with now [46]. Finally, a qualitative study of older adult DOC users suggests that the DOC provides a consistent source of support even when someone’s physical location may change [41]. As older adult social networks get smaller because of death and relocation, the DOC may be one for maintaining social connections and avoiding isolation while supporting health.

DOC users find the information found on the DOC to be credible, overall. However, we found that DOC users found information from their health care providers to be more competent and trustworthy than the information found on the DOC. In contrast, research specific to 1 type of DOC focused on patient-driven diabetes innovation, with membership of mostly individuals with type 1 diabetes, found that peers were reported to be more trustworthy than health care providers [47]. Perhaps, this can be explained by the difference between a general DOC and a specialty DOC or the presence of various types of diabetes that we studied compared with type 1 only.

DOC source credibility was associated with high diabetes self-care and high social capital. DOC users were able to validate their experiences through homogenous DOC users, while gaining diverse information from heterogeneous DOC users to improve self-care. Obtaining these different perspectives on diabetes care provides DOC users with more depth of knowledge when making their own health care decisions [41] and supports patient activation [48]. Although we did not seek out information specific to the presence of misinformation, which can impact a DOC user’s perception of source credibility, other research has found that misinformation in the DOC is uncommon and corrected by peers in the DOC or benign when it does occur [33,49-51].

Interestingly, DOC users who felt supported by their health care provider to use the DOC found the information on the DOC to be more credible and helpful. This suggests that health care providers play a role in how DOC users perceive DOC source credibility and should engage in the DOC, as recommended by Brady et al [52], to understand the resources available to people with diabetes. A 2017 American Association of Diabetes Educator National Practice survey found that 34.7% of diabetes educators are recommending DOC sources to their patients and nearly three-quarters (73.4%) are using the DOC themselves in some way [53]. Although there is evidence that diabetes educators seem to be embracing DOCs, it is unknown if other health care providers, including those who routinely care for individuals with diabetes, are actively or passively participating in DOCs.

DOC users have high bonding and bridging social capital scores. Those who felt more connected to the DOC reported greater benefits with regard to knowledge attainment, social support, and empowerment. Those with high bridging social capital also had high diabetes self-care scores. Perhaps, this can be explained by the information gained from individuals who may have different diabetes experiences and treatment regimens that provide sources of education. One study found that 76% of DOC users learned new diabetes management strategies from their peers [7]. In another study, when compared with non-DOC users, DOC users engaged in more self-care activities related to healthy diet, exercise, checking glucose, and taking insulin [54]. This study also indicates that individuals are able to learn things that their health care providers did not know through social capital.

Social capital provides a sense of social connectedness. Putnam [22] found that social connectedness strongly predicts altruism. Altruism has been identified in other online communities [6,28,55,56]. Individuals who engage in the DOC may be providing emotional, informational, instrumental, or companionship support to other DOC users for several reasons. DOC users may want to prevent others from experiencing any hardships they may have encountered, as such, they provide information to support learning. Conceivably, altruism comes full circle in that the support an individual DOC user provides the community is reciprocated in ways that benefit the individual DOC user in some way. For example, a baby boomer DOC user transitioning into retirement may find meaning and purpose in supporting other DOC users, which has been associated with improved physical function [57].

Overall, the DOC was seen as helpful and study participants reported minor harm only in a few instances. The findings were similar to those of a national survey of the general population [29] and another specialty DOC study [47]. There were marked differences in participants who were not sure if they were helped or harmed by the DOC, which warrants further study. Although additional information about harm was not asked in this study, our findings, and findings of others [47] who explored help and harm in a similar way, suggest that DOC use is beneficial with low risk. It is important to note that online peer health may not be helpful for all individuals with diabetes [58]. A secondary factor that unites peers, such as gender, culture, age, or shared experience [59], which is available within the DOC, may be necessary for optimal outcomes.

In summary, individuals seek online health information to fill a gap in their health care needs. The DOC appears to fill a void in the current health care system with regard to day-to-day support [6,7,54,60] and is perceived as credible and helpful. Health care providers need to understand that although they are key sources for health information, they are among a large network of potential health information sources [61,62], which may include family, friends, and online peers with a similar condition. Access to social support, which has been identified within the DOC, can mediate better health outcomes [63] for baby boomers and younger adults.

### Limitations

The sample was overwhelmingly white and living in the United States, which may not be representative of the entire DOCs studied. Furthermore, this study examined only those who could read English. Individuals engaged in non-English speaking DOC sites (ie, EstaTuDiabetes) may elicit different results. The DOC source credibility measured a collection of information from the participant’s interaction with DOC users as a whole when in fact a DOC user may rely on information from select individuals and avoid information from others. For those individuals who reported harm, it is unknown if that harm caused physical or mental harm or another form of harm. Finally, because of self-selection, generalizations should not be made.

### Conclusions

This is the first study to identify how DOC users view source credibility specifically to the DOC and their diabetes health care providers, social capital, and help and harm from the DOC. Baby boomers and younger adults perceived the credibility of DOC information similarly and found DOC use to be beneficial with low risk. DOC users found their health care providers to be more competent and trustworthy compared with the DOC, suggesting that DOC users find their health care provider valuable, despite their DOC use. Furthermore, a randomized clinical trial with DOC-naïve participants is warranted to understand the impact of DOCs on health outcomes, including variations of help and harm.

## Abbreviations

HbA_1c_: glycosylated hemoglobin
DOC: diabetes online community
LADA: latent autoimmune diabetes of adulthood
REDCap: Research Electronic Data Capture
SCI-R: Self-Care Inventory Revised
